# Supplementary material for: The Quality and Fertilizing Potential of Red Deer (Cervus elaphus L.) Epididymal Spermatozoa Stored in a Liquid State
Source: Int J Mol Sci. 2022 Nov 23;23(23):14591. doi: 10.3390/ijms232314591 (PMC9741111; doi:10.3390/ijms232314591)
Supplement: Supplementary file 1 [file ijms-23-14591-s001.zip › Supplementary Table S1.pdf]

Table S1. Motility parameters of red deer epididymal spermatozoa (*n* = 5) stored in a liquid state at 5 °C that were used for the in vitro fertilization of oocytes and artificial insemination of hinds.

Sperm quality parameters	Storage time (days)				
	Day 1	Day 5	Day 7	Day 10	Day 11
TMOT (%)	82.6 ± 1.7^{a}	71.8 ± 3.7^{ab}	71.0 ± 4.1^{ab}	64.8 ± 3.7^{b}	62.8 ± 2.3^{b}
PMOT (%)	42.8 ± 3.3^{a}	34.6 ± 6.3^{ab}	34.8 ± 4.2^{b}	21.6 ± 4.5^{c}	12.2 ± 0.8^{c}
VAP (µm/s)	131.1 ± 4.1^{a}	102.2 ± 7.3^{b}	99.1 ± 3.9^{b}	93.8 ± 5.1^{b}	84.9 ± 3.6^{b}
VSL (µm/s)	100.7 ± 3.9^{a}	79.5 ± 7.6^{b}	77.1 ± 3.8^{bc}	65.1 ± 5.9^{c}	54.3 ± 0.7^{c}
VCL (µm/s)	222.5 ± 6.5^{a}	182.7 ± 11.5^{a}	175.1 ± 10.2^{a}	178.9 ± 14.3^{a}	170.0 ± 8.3^{b}
ALH (µm)	7.6 ± 0.2^{a}	7.3 ± 0.1^{a}	6.9 ± 0.4^{a}	7.4 ± 0.7^{a}	7.5 ± 0.1^{a}
BCF (Hz)	38.7 ± 0.2^{a}	35.8 ± 2.2^{a}	37.4 ± 1.3^{a}	34.1 ± 1.9^{b}	32.1 ± 0.5^{b}
STR (%)	74.6 ± 1.1^{a}	75.0 ± 2.6^{ab}	74.6 ± 2.2^{ab}	67.2 ± 5.1^{b}	61.8 ± 2.0^{b}
LIN (%)	46.2 ± 1.8^{a}	43.4 ± 2.1^{ab}	44.8 ± 2.4^{ab}	38.2 ± 4.2^{b}	32.8 ± 1.4^{b}

TMOT, total motility. PMOT, progressive motility. VAP, velocity average path. VSL, velocity straight line. VCL, curvilinear velocity. ALH, mean amplitude of lateral head displacement. BCF, beat cross frequency. STR, straightness. LIN, linearity. Values are expressed as means ± SEM. $^{a\text{-}c}$ Values within a row with different superscripts differ significantly at $p < 0.05$.
