# Supplementary material for: The Quality and Fertilizing Potential of Red Deer (Cervus elaphus L.) Epididymal Spermatozoa Stored in a Liquid State
Source: Int J Mol Sci. 2022 Nov 23;23(23):14591. doi: 10.3390/ijms232314591 (PMC9741111; doi:10.3390/ijms232314591)
Supplement: Supplementary file 1 [file ijms-23-14591-s001.zip › Supplementary Table S2.pdf]

Table S2. Quality parameters of red deer epididymal spermatozoa ($n = 5$) stored in a liquid state at 5 °C that were used for the *in vitro* fertilization of oocytes and artificial insemination of hinds.

Sperm quality parameters	Storage time (days)				
	Day 1	Day 5	Day 7	Day 10	Day 11
MOR (%)	71.9 ± 1.3^{a}	62.2 ± 2.9^{b}	63.2 ± 3.8^{b}	64.5 ± 3.9^{bc}	56.0 ± 3.7^{c}
NAR (%)	84.8 ± 2.2^{a}	81.8 ± 1.6^{ab}	82.7 ± 2.2^{ab}	84.0 ± 2.0^{b}	79.5 ± 1.6^{b}
HD (%)	11.5 ± 1.5^{a}	14.5 ± 0.8^{ab}	14.0 ± 0.8^{ab}	15.4 ± 1.7^{ab}	15.5 ± 1.5^{b}
MD (%)	1.8 ± 0.3^{a}	2.2 ± 0.6^{a}	2.5 ± 0.7	1.0 ± 0.2^{a}	1.5 ± 0.3^{b}
TD (%)	7.5 ± 1.2^{a}	15.7 ± 4.4^{ab}	15.1 ± 4.7^{ab}	14.2 ± 3.7^{b}	23.6 ± 5.6^{b}
PNA^{-}/PI^{-} (%)	76.2 ± 3.4^{a}	71.2 ± 1.8^{ab}	67.5 ± 2.2^{ab}	60.5 ± 3.8^{b}	61.6 ± 5.1^{b}
$SYBR^{+}/PI^{-}$ (%)	90.5 ± 0.1^{a}	86.0 ± 2.0^{ab}	86.0 ± 3.2^{ab}	82.0 ± 1.6^{ab}	80.2 ± 1.3^{b}
MMP (%)	93.1 ± 2.0^{a}	90.1 ± 2.4^{ab}	88.1 ± 3.4^{ab}	81.2 ± 4.1^{b}	78.7 ± 3.3^{b}
AO (%)	94.9 ± 1.2	91.5 ± 3.4	94.3 ± 1.8	93.4 ± 0.7	89.2 ± 1.6
$YOPRO^{-}/PI^{-}$ (%)	88.2 ± 2.0^{a}	67.0 ± 3.0^{b}	66.1 ± 2.5^{b}	67.8 ± 2.2^{b}	59.7 ± 4.2^{b}
$YOPRO^{+}/PI^{-}$ (%)	3.5 ± 1.2^{a}	15.8 ± 6.3^{b}	15.5 ± 6.5^{b}	12.4 ± 4.3^{b}	18.4 ± 5.7^{b}

MOR, normal sperm. NAR, normal apical ridge acrosomes. HD, head defects. MD, midpiece defects. TD, tail defects. PNA^{-}/PI^{-}, acrosomal status. $SYBR^{+}/PI^{-}$, viability. MMP, mitochondrial membrane potential. AO, DNA integrity. $YOPRO^{-}/PI^{-}$, viable, non-apoptotic sperm. $YOPRO^{+}/PI^{-}$, apoptotic sperm. Values are expressed as means ± SEM. Differences between means were determined by Student Newman–Keuls post hoc test. $^{a-c}$ Values within a row with different superscripts differ significantly at $p < 0.05$.
